# Ultrasound-assisted catheter-directed thrombolysis in a patient with COVID-19 infection and bilateral intermediate-to-high-risk pulmonary embolism: a case report

**DOI:** 10.1093/ehjcr/ytad628

**Published:** 2024-01-08

**Authors:** Grigorios Korosoglou, Dimitrios Mouselimis, Elke Koenig, Stavros Konstantinides

**Affiliations:** Department of Cardiology, GRN Hospital Weinheim, Vascular Medicine & Pneumology, Röntgenstrasse 1, 69469 Weinheim, Germany; Weinheim Imaging Center, Hector Foundation, Röntgenstrasse 1, 69469 Weinheim, Germany; Department of Cardiology, GRN Hospital Weinheim, Vascular Medicine & Pneumology, Röntgenstrasse 1, 69469 Weinheim, Germany; Weinheim Imaging Center, Hector Foundation, Röntgenstrasse 1, 69469 Weinheim, Germany; Department of Anesthesiology and Intensive Care Medicine, GRN Hospital Weinheim, Weinheim, Germany; Center for Thrombosis and Hemostasis, University Medical Center Mainz, Mainz, Germany; Department of Cardiology, Democritus University of Thrace, Alexandroupolis, Greece

**Keywords:** Case report, Intermediate-to-high-risk pulmonary embolism, Ultrasound-assisted catheter-directed thrombolysis, Right ventricular dilatation, Computed tomography angiography, COVID-19

## Abstract

**Background:**

Acute pulmonary embolism (PE) is a common cardiovascular disorder, potentially associated with high morbidity and mortality rates.

**Case summary:**

Herein, we report on a patient with COVID-19 infection and bilateral PE, who presented after cardiovascular resuscitation with return of spontaneous circulation. Initially, an acute coronary syndrome was suspected but bedside echocardiography showed dilatation of the right ventricle (RV) and RV dysfunction, helping to establish the diagnosis of acute intermediate-to-high-risk PE, which was subsequently confirmed by contrast-enhanced computed tomography pulmonary angiography. The patient was successfully treated using low-dose (12 mg of tissue plasminogen) ultrasound-assisted catheter-directed thrombolysis, which resulted in prompt clinical improvement and reversal of RV dysfunction without bleeding complications.

**Discussion:**

This case demonstrates the importance of echocardiography for the differential diagnosis of PE and of catheter-directed thrombolysis for its treatment in patients with intermediate-to-high-risk and high-risk PEs.

**Learning points**

- Acute pulmonary embolism (PE) is a common cardiovascular disorder, potentially associated with high morbidity and mortality rates. COVID-19 may increase the risk for PE.
- The role of ECG (precordial leads V1R to V6R), echocardiography, and biomarkers is crucial for the diagnosis of acute coronary syndromes vs. PE.
- Catheter-directed thrombolysis may be a potential lifesaving treatment strategy in patients with intermediate-high and high-risk PEs.

## Introduction

Pulmonary embolisms (PEs) are one of the most frequent acute cardiovascular disorders with an annual incidence of 39–115 per 100 000 individuals worldwide, leading to excess morbidity and mortality both in Europe and in the USA.^1–3^ Especially during the SARS-CoV-2 pandemic, the incidence of thrombosis and associated complications was high in patients with COVID-19 infection, particularly affecting critically ill patients, who required hospitalization in intensive care units.^4^

Systemic fibrinolysis has the potential to reduce acute right ventricular (RV) pressure overload in high-risk (massive) and intermediate-to-high-risk (submassive) PEs, resulting to lower mortality rates by reversal of pulmonary artery obstruction and RV failure.^3,5,6^ However, full-dose systemic fibrinolysis, although effective in reversing RV failure, is associated with 5- to 10-fold increase of major bleeding and haemorrhagic stroke,^7^ so that this treatment is primarily restricted to patients with persistent haemodynamic instability due to PE and not to intermediate-to-high-risk patients.^3^

In search of less aggressive fibrinolytic treatment regimes, low-dose catheter-directed fibrinolytic therapy has been introduced as an option for pharmaco-mechanical thrombus dissolvement in intermediate-to-high-risk PE.^8^ Herein, we present the case of a patient with COVID-19 infection and bilateral acute PE with initial haemodynamic instability, who was treated using ultrasound-assisted catheter-directed thrombolysis.

## Summary figure

**Table:** 

Time points	Events
Initial presentation	55-Year-old male patient with flu-like symptoms and fever during the last 2 weeks was admitted after out-of-hospital resuscitated cardiac arrest with ROSC. The patient had dyspnoea and chest pain. The clinical findings included tachycardia, hypoxia, ST-segment depression in V1–V3, and negative T-waves in V4–V6 but no ST-elevation. In addition, a positive rapid COVID-19 test, increased hs-TnT, and echocardiographic signs of right ventricular dysfunction were present. Pulmonary embolism (PE) was suspected, which was confirmed by computed tomography angiography (CTA).
Intervention (Day 0)	The patient was directly transferred to intensive care unit and ultrasound-assisted catheter-directed thrombolysis was performed during the same day, using the EkoSonic Endovascular System. Heart rate, blood pressure, and O_2_ saturation gradually improved.
Day 1	O_2_ supply was not necessary any more, hs-TnT decreased, and an almost complete resolution of RV enlargement was observed by echocardiography. Rivaroxaban 15 mg twice daily was initiated, and the patient was transferred to the normal ward.
Day 2	The patient was discharged and repeated CTA was planned due to moderate calcification of the coronary arteries and to judge the amounts of remaining thrombus after catheter-directed thrombolysis.
Day 16	In the coronary CTA, a normalization of the RV diameter and only small residual thrombi in the pulmonary arteries were detected. Obstructive coronary artery disease was not present.
Three months follow-up after Day 16 only 5 months of follow-up	The further clinical course was uneventful.

## Case presentation

A 55-year-old male patient was admitted to our emergency department after mechanical out-of-hospital cardiovascular resuscitation (20–30 s) with almost immediate return of spontaneous circulation (ROSC). On admission, the patient reported persisting chest pain and shortness of breath. In addition, he reported fever with flu-like symptoms during the past 2 weeks. No cardiovascular risk factors were present, and the patient had no history of coronary artery disease, heart failure or prior myocardial infarction.

Clinical examination revealed tachycardia with a heart rate of 120 b.p.m., low blood pressure of 100/60 mmHg without mechanical circulatory support or infusion of catecholamines, and blood oxygen saturation level of 90% despite oxygen delivery (6 L/min). The ECG exhibited ST-segment depression in V1–V3 and negative T-waves in V4–6. ST-elevations were not present, including the precordial leads V1R to V6R. In addition, the rapid COVID-19 test was positive and PCR confirmed COVID-19 infection, with a cycle threshold value of 33. Laboratory testing showed increased C-reactive protein (16.4 mg/L, reference value lower than 5 mg/L), white blood count (11.4/nL), and high-sensitive troponin T (hs-TnT) of 126 ng/L (reference value lower than 14 ng/L). Non-ST-elevation myocardial infarction was suspected, and the patient was initially scheduled for coronary angiography.

However, bedside echocardiography revealed dilatation of the right ventricle (RV) causing RV dysfunction and D-shape of the left ventricle. Left ventricle contractility was normal without global or regional wall motion abnormalities (ejection fraction of 65%). In the absence of inferior wall motion abnormalities and precordial ST-elevation in V1R to V6R, acute PE turned out to be the most probable diagnosis. D-Dimer was also increased (4550 µg/L, reference value lower than 500 µg/L). In addition, no signs of DVT were present at admission of the patient who exhibited a Wells score of 4.5 points, PESI score of 155 points,^9–10^ and simplified PESI score of 3 points.^11^

**Figure 1:**
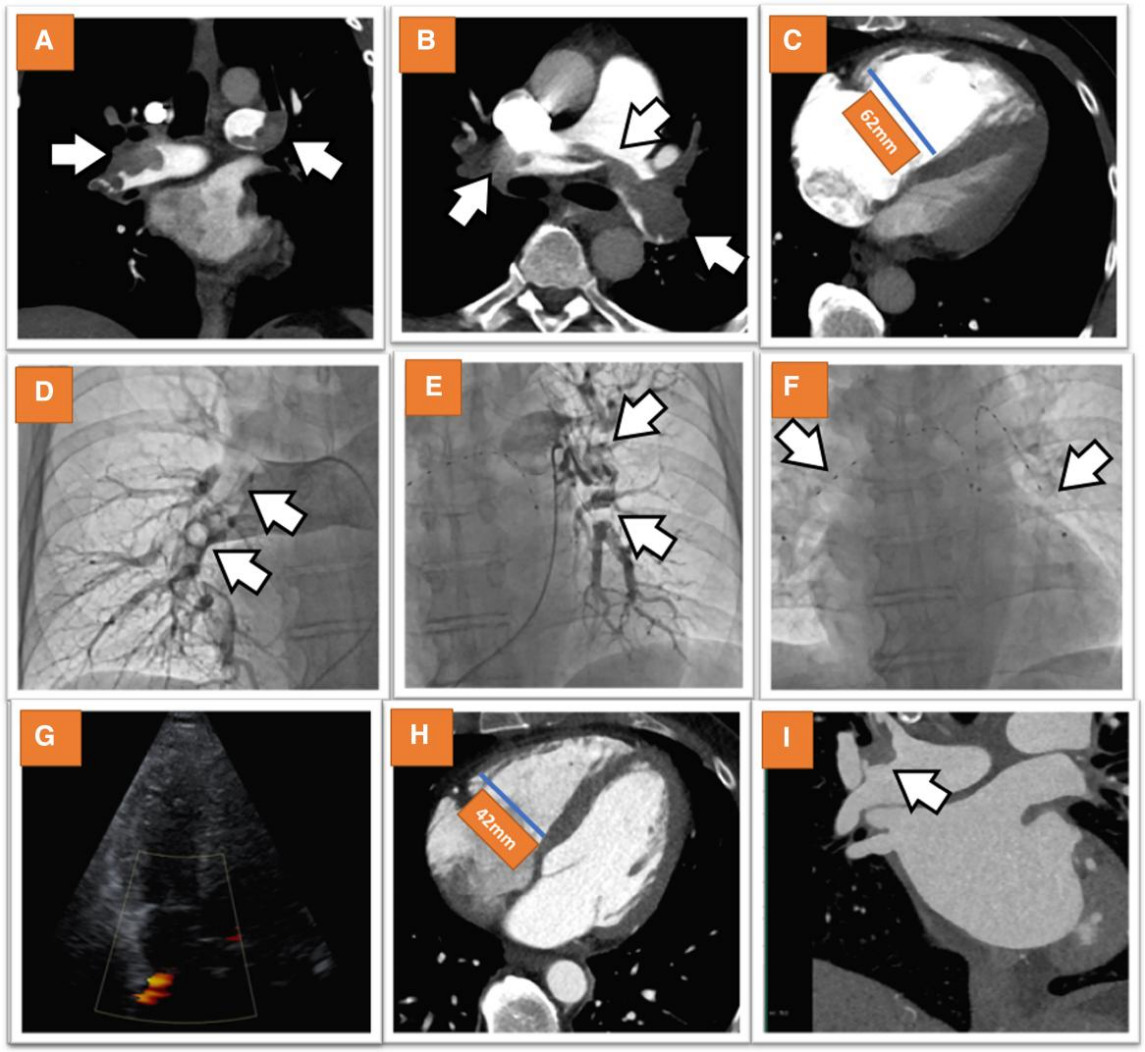
CTA showed massive, bilateral acute PE (*A* and *B*) and massive dilatation of the RV (*C*). Pulmonary angiography confirmed massive bilateral PE (*D* and *E*) and two EKOS catheters were inserted in the right and left pulmonary arteries under fluoroscopic control (*F*). Echocardiography on the next day demonstrated complete resolution of RV enlargement with normal RV diameter of 42 mm and function (*G*). Control CTPA after 2 weeks demonstrated normalization of the RV diameter (*H*) and small amounts of residual thrombi in the pulmonary arteries (*I*).

Contrast-enhanced computed tomography pulmonary angiography (CTPA) confirmed the presence of massive, bilateral pulmonary emboli (*Figure 1A* and *B*), also confirming the dilatation of the RV with a diameter of 62 mm at baseline (*Figure 1C*). Thus, the diagnosis of intermediate-to-high-risk acute PE was established, the patient was transferred to our intensive care unit, and percutaneous catheter-directed thrombolysis was scheduled for the same day. After puncture of the right femoral vein, two 7 F sheaths were inserted to facilitate bilateral ultrasound-assisted catheter-directed thrombolysis, using the EkoSonic Endovascular System (EKOS, Boston Scientific, MN, USA). Pulmonary angiography confirmed massive bilateral PE (*Figure 1D* and *E*) and two EKOS catheters were inserted into the right and left pulmonary arteries, respectively (*Figure 1F*, Supplementary material online, Video*S1*). Mean pulmonary artery pressure was measured at 35 mmHg (70 and 25 mmHg systolic and diastolic pressure, respectively). Local thrombolysis was performed with tissue plasminogen (t-PA) at an infusion rate of 1 mL/h over 6 h bilaterally. In addition, unfractionated heparin was infused, targeting an activated partial thromboplastin time of 50–70 s. A total dose of 12 mg t-PA was administrated. The course during local lysis was uneventful and the patient’s blood pressure and oxygen saturation levels gradually increased, while his heart rate decreased. On the next day, the oxygen saturation reached a level of 98% without oxygen supply, his blood pressure was 130/80 mmHg, and his heart rate was 80 b.p.m. Cardiac troponin levels decreased to 98 ng/L and bedside echocardiography demonstrated almost complete resolution of RV enlargement with a RV diameter of 42 mm and function (*Figure 1G*, Supplementary material online, Video*S2*). Systolic pulmonary artery pressure was estimated at 35 mmHg by echocardiography. The patient was transferred to the normal ward, and oral anticoagulation treatment was started with rivaroxaban 15 mg twice a day. The further clinical course was uneventful, and the patient was discharged on the following day. Repeat CTPA after 2 weeks, which was performed due to moderate calcification of the coronary arteries and to determine the effectiveness of lytic therapy and subsequent anticoagulation and the amounts of residual thrombi, demonstrated normalization of the RV diameter (42 mm) and only small residual thrombi in the pulmonary arteries (panels *H*–*I*). The patient continued treatment with 20 mg rivaroxaban once daily after 3 weeks of 15 mg rivaroxaban twice per day, and his further course during the next 5 months is still uneventful. Rivaroxaban 20 mg once per day was decided to be continued up to 6 months after the index hospitalization of the patient, followed by 10 mg rivaroxaban lifelong. In addition, malignancy was excluded by additional abdominal ultrasound, gastroscopy, and colonoscopy.

## Discussion

Catheter-directed thrombolysis has been increasingly used in patients with intermediate-to-high-risk and high-risk PEs for RV failure reversal, emerging as a potential lifesaving treatment strategy.^8,12^ Its use increased during the last years after randomized trials and meta-analyses pointed out that systemic fibrinolytic treatment is associated with mortality benefits at the cost of life-threatening bleeding complications.^13^ Thus, since catheter-directed thrombolysis uses only a fraction of the systemic lytic dose (total t-PA dose of 20–24 mg), which is given over a longer time frame, less bleeding complications are anticipated, and this is supported by the results of randomized trials and registries.^8,14,15^ Especially with the EKOS system, the high-frequency ultrasound component of the catheter-directed fibrinolysis is postulated to further disaggregate fibrin fibres, potentially allowing greater penetration of the fibrinolytic agent into the thrombus despite the low dose of the applied thrombolytic agents.^16^

In our case, the EKOS system was successfully used in a patient with COVID-19 infection and intermediate-to-high-risk PE. The exact pathogenesis of hypercoagulability and thrombosis with COVID-19 patients, addressed as COVID-19 associated coagulopathy, is not yet fully understood.^17^ Hypercoagulation, immune-response associated inflammation, and endothelial injury are potentially linked to this disorder.^18,19^ Generally, PE needs to be considered in case of haemodynamical instability or deteriorating gas exchange in patients hospitalized for COVID-19 disease.^20^ Our patient, however, was not hospitalized for COVID-19 infection but exhibited ambulatory flu-like symptoms, which initially did not require hospitalization. Thus, beyond viral infection, other causes for PE such as malignancy need to be considered in such patients. Furthermore, antiphospholipid assessment needs to be considered in patients with intermediate-to-high-risk and high-risk PEs since patients with clinical symptoms of an antiphospholipid syndrome and with triple positivity with Lupus anticoagulant, anticardiolipin-antibodies (ACA) and β2-glycoprotein I-antibodies (β2GPI-Ab) exhibit increased risk of arterial thromboembolic events during treatment with new oral anticoagulants (NOAC) and should therefore be treated with vitamin K antagonists. NT-pro-BNP values were not available with our patient, which is a limitation, due to the prognostic relevance of this biomarker in patients with PE.

In addition, the clinical presentation including chest pain, ROSC, and ECG abnormalities was suggestive for acute coronary syndrome rather than PE. This underscores the role of clinical examination, the ECG, and bedside echocardiography in such patients, which in that case immediately helped to establish the correct diagnosis and initiate the next therapeutic steps. Bilateral ultrasound-assisted catheter-directed thrombolysis was successfully performed, using only 12 mg of t-PA, which resulted in prompt resolution of clinical symptoms and reversal of RV failure during the first 24 h of treatment, without bleeding complications.

In conclusion, emerging technical developments allow for low-dose ultrasound-assisted catheter-directed thrombolysis. Such an approach may be feasible in patients with intermediate-to-high-risk and high-risk PEs.

## Supplementary Material

ytad628_Supplementary_Data

## Data Availability

The data that support the findings of this study are available from the corresponding author upon reasonable request.

## Lead author biography

Professor Grigorios Korosoglou has been chair of the Departments of Cardiology, Vascular Medicine, and Pneumology in the GRN Academic Teaching Hospital Weinheim since 2015. Since 2022 he is chair of the Departments of Cardiology and Vascular Medicine in the GRN Academic Teaching Hospitals Weinheim and Eberbach.

## Supplementary material

**Consent**: The authors confirm that written informed consent for submission and publication of this case report including images and associated text has been obtained from the patient in line with COPE guidelines.

**Funding:** Not applicable.
